# A Workplace Environmental Scan of Employed Carers During COVID-19

**DOI:** 10.1007/s10834-023-09898-9

**Published:** 2023-05-24

**Authors:** Regina Ding, Jenny Ploeg, Allison Williams

**Affiliations:** 1 School of Earth, Environment, and Society, McMaster University, 1280 Main St W, Hamilton, ON L8S 4L8 Canada; 2 Present Address: Sprott School of Business, Carleton University, 1125 Colonel By Drive, Ottawa, ON K1S 5B6 Canada; 3 School of Nursing, McMaster University, Hamilton, Canada

**Keywords:** Caregiving, Workplace, COVID-19, Work-life conflict

## Abstract

The carer-employee experience has undergone multiple shifts during the COVID-19 pandemic. This study seeks to understand how changes in the workplace as a result of the pandemic have impacted employed carers with their ability to perform both care obligations and paid work responsibilities. Using an online workplace-wide survey at a large Canadian firm, we conducted an environmental scan of: the current state of workplace supports and accommodations, supervisor attitudes, and carer-employee burden and health. Our findings demonstrate that while employees are generally in good health, care burden and time spent caregiving has been higher during COVID-19. Notably, employee presenteeism is higher during the pandemic than it was previously, with carer-employees experiencing significantly reduced levels of co-worker support. The most common workplace adaptation to COVID-19, work-from-home, was preferred by all employees as it allowed greater schedule control. However, this comes at the cost of reduced communications and sense of workplace culture, especially for carer-employees. We identified several actionable changes within the workplace, including: greater visibility of existing carer resources, and standardized training of managers on carer issues.

## Introduction

The World Health Organization (WHO) formally declared COVID-19 a pandemic in March 2020. However, well before its official classification, the virus had already disrupted the lives and routines of millions around the world, and continues to influence many radical shifts across all corners of life. Undeniably, the COVID-19 pandemic had rapidly become the instigating force of many small and large scale transformations, from our activities of daily living to the global socio-political stage. One of the most prominent reimaginings taking place in our daily lives, is the way that employees experience and carry out paid work, causing additional strain on workers who are involved care provision for family and friends.

On a global scale, physical distancing mandates required many businesses to convert to virtual or remote operations. While originally conceived as a temporary measure to quickly reduce the infectivity of the COVID-19 virus, this period of altered work quickly become the norm for the months and years after the WHO’s declaration of the COVID-19 pandemic, with the speculation that some workplaces would continue to operate remotely post-COVID (Bick et al., 2021). Generally, the research on remote work (sometimes known as telework) prior to COVID has been positive for employers and employees; with remote work associated with increases in employee work performance, reduced turnover, and worker stress (Golden, 2006; Hunter, 2019; Mann & Holdsworth, 2003). Within the past decade in particular, flexible work arrangements, of which telework and remote working is included, have been held as the gold standard for reconciling work-life balance issues (Azar et al., 2018; Kossek & Ozeki, 1999).

The COVID pandemic has been a catalyst in promoting the discourse around remote work and work-life balance into public view, particularly as it pertains to eldercare. Given the increased vulnerability of seniors and the immunocompromised to COVID, public attention turned to not only the protection of these vulnerable groups against illness, but also those responsible for their care. In Canada, the combination of an aging population coupled with the prevalence of community-based care in the aftermath of the neoliberal reforms in the 1980s mean that carers make up a significant proportion of society, and an even greater proportion of the labour force (Fast et al., 1999). In the context of COVID, carers, particularly those active in the labourforce, have become a salient population to take heed of, as they navigate the conflicting responsibilities of paid work and care provision during COVID. In particular, the nature of remote working introduces several challenges in the way that work is performed that may negatively interact with other personal responsibility, especially caregiving within the wake of COVID. In this way, workplaces may be recognized as salient arenas in which both family and work issues may be addressed simultaneously.

## Literature Review

A changing demographic landscape was already underway prior to the arrival of COVID, with changing labour force needs. Canada is currently classified as an aging nation, with approximately 18.4% of the population over the age of 65, with projections of continued progressive aging in the near future (Statistics Canada, 2021). This pattern of age distribution has wide-scale ramifications for employers in multiple ways. First, the available labour supply shrinks as older workers retire and there are fewer available younger workers to replace them due to declining fertility rates. Secondly, as workers themselves age and progress in their careers, they are more likely to be involved in the care provision of their aging relatives. As of 2018, there were 7.8 million carers in Canada (approximately 1 in 4 Canadians), with 6.1 million of these carers also engaged in the labour force (Employer Panel for Caregivers, 2015; Statistics Canada, 2018). While historically, the carer role fell onto women, the increasing labour force participation of women and rise in dual-income households point to a more balanced distribution of care responsibility, with 54% of carers identifying as women and 46% as men (Sinha, 2012). The responsibility of being a carer to family and friends often conflicts with paid employment responsibilities, leading to tensions in both roles.

Carer-employee burden is defined as the strain and role conflicts associated with the simultaneous management of both paid work obligations and unpaid care provision to family and/or friends (Ding et al., 2020). This burden known to be both dynamic and bi-directional, with paid work impacting on caregiving and vice versa. From previous research, it was observed that when carer and worker roles conflict, paid work responsibilities often take priority over caregiving, regardless of carer-employees’ own preferences (Ding & Williams., 2022). This type of conflict, known generally as work-family conflict, is associated with decreased job satisfaction and increased job turnover, role stress, and burnout (Boles et al, 2001; Marks, 1998). Conflicts which result in caregiving taking precedence over paid work, known as family-work conflict, are understudied in comparison to work-family conflict, although there is some evidence that adverse consequences may manifest in both the work and home domains (Boles et al, 2001).

When unaddressed, carer-employee burden is costly to employers. In Canada, it is estimated that $1.3 billion worth of productive work is lost annually due to caregiving impacts on paid work, leading to absenteeism and turnover (Employer Panel for Caregivers, 2015). Approximately 40% of carer-employees reported disruptions to their work schedules (i.e., arriving late, taking time off) due to caregiving responsibilities, with 15% reducing weekly hours of work to accommodate caregiving (Sinha, 2012). From the US, a national survey (N = 4335) found that being a carer to an adult with health issues increased risk of wage loss by 29%, with each additional hour spent per week on care provision increasing this risk by 3% (Earle & Hayman, 2012). Dumont et al. (2015) found that over a 101-day observational window, carers to palliative care recipients missed on average 32.35 and 41.42 h of work due to caring responsibilities in urban and rural areas respectively. Other non-easily monetized consequences of carer-employee burden include increased stress, anxiety, depression, job turnover, and decreased social connections, life satisfaction, and career progression (Schultz & Sherwood, 2008).

This dynamic has worsened during the COVID-19 pandemic; not only have there been large-scale deviations to our everyday mobility and locations of work and care, but older adults and those with prior health issues are most vulnerable to severe inflection by the virus, leading to heightened anxieties for carers. Previously, carers of older adults, on average, spent 17 h per week on caregiving (CIHI, n.d). However, the pandemic has intensified care demands due to loss of social supports, respite services, and healthcare access; approximately 56% of surveyed carers in Manitoba indicated that formal support services for their care recipient had been suspended during COVID (Funk et al., 2021). Emerging research found that 60% of carers were providing more weekly care hours during the pandemic compared to before (Funk et al., 2021). A national survey from the UK found that 81% of carers were providing more care during the pandemic than prior to, with 58% of carers reporting the associated stress of caregiving during the pandemic as having negative impacts on their own health and wellbeing (Carers, 2020). A Canadian longitudinal survey found that participants reported significantly higher depressive symptoms as the pandemic continued; this was particularly true for female carers, who reported higher depression and anxiety symptoms than their male carer counterparts (Wister et al., 2022). Similarly, in Alberta, 70% of surveyed carer-employees in the healthcare sector reported worse mental health during the pandemic compared to before, and 50% reported financial difficulty (Caregiver Centered Care, 2021). In addition to changing care landscapes, the work experience was also radicalized, with furloughs, lay-offs, physical distancing, uptake of personal protective equipment (PPE), and pivot to remote working widespread. These changes are accompanied by worsening employee mental health, and increased financial difficulties, and job anxiety (Hamouche, 2020). Although research on carer-employee experience during COVID is sparse, it is conceivable that carer-burden has significantly increased during COVID as a result of shifts in the work-care dynamic.

Workplaces can play pivotal roles in mitigating carer-employee burden. As of 2017, the Canadian Standards Association (CSA) published a workplace standard on how employers can and should support their employees who are caregiving, signaling that there is an increasing spotlight on workplaces and their role in supporting carers (CSA, 2017). Supportive workplace culture is a well-known buffer to adverse consequences of work-family conflict, with some scholars suggesting that informal systems of support play a more crucial role than formal supports at influencing job satisfaction, stress, caregiver stress, turnover and absenteeism (Behson, 2005; Miller et al., 2001). Work-family conflict was found to be reduced by employee access to schedule flexibility, family-friendly supervisors, and supportive coworkers (Hammer et al., 2011). Conceptual models theorize that supportive workplaces form an organizational impetus for supervisors and coworkers to decrease work interference on family, leading to reduced job turnover and higher commitment (Ahmad & Omar, 2010; Fiksenbaum, 2014; Kossek et al., 2010). The presence of a family supportive supervisor at work reduces the risk of carer-employee wage loss by 37%, with access to paid leave providing a 30% reduction in risk (Earle & Heymann, 2012). With COVID, carers’ stress process is likely to be disrupted as secondary stressors such as paid work are amplified and may lead to decline in wellbeing (Schmitz et al., 2022,1). Given this, it is within the best interests of workplaces to consider implementing carer-friendly practices, as workplace needs of economically active Canadians will continue to shift in the coming years.

The current literature on caregiving and paid employment however, is lacking in how the work landscape has changed with COVID, and how carer-employees are adapting their work and care responsibilities. This knowledge is necessary as an appropriate workplace baseline must be established so that (1) future interventions have a local context in which they operate within and; (2) interventions can be tailored to the needs and gaps of a specific workplace. For these reasons, an environmental scan is necessary within each workplace prior to intervention design and implementation.

We partnered with a large (4000 + employees) Canadian engineering firm in the oil and gas industry to conduct an environmental scan of the workplace experience during COVID-19, with particular emphasis on carer-employees and their experience balancing informal caregiving with paid work obligations. This scan will serve as the organization-specific baseline measurement and context for workplace culture, health, employee satisfaction and COVID changes. A workplace intervention will be designed and implemented, building on the findings from this environmental scan, with the aim of increasing carer-employee supports within this particular worksite.

We set out to answer the following questions:

- OBJECTIVE 1: In what ways has the paid work experience changed with COVID-19?
- OBJECTIVE 2: Do the work and health outcomes of carer-employees differ significantly from non-carer/regular employees?
- OBJECTIVE 3: How can workplaces adapt to support their employees, caregiving or otherwise?

## Methods

This study follows an explanatory sequential structure, where quantitative data was analyzed first, followed by qualitative data used to generate in-depth meaning and understanding. The study was approved by university ethics (MREB 2434) during the spring of 2020, after the onset of the COVID-19 pandemic. During the spring of 2020, a call for workplaces was distributed electronically and word-of-mouth through: partnered universities and research networks, conferences, national and provincial carer networks and non-profits. Eligibility criteria was solely that the workplace was interested in promoting a carer-friendly workplace, with no restrictions on workplace size or industry. One suitable workplace was identified and recruited after referral by Carers Canada. Several virtual meetings were held with senior executives to fully convey the nature of the research and expected collaboration. The workplace – the Canadian division of a multinational engineering consulting group—was formally recruited in June of 2020 after obtaining a signed letter of consent from the Vice-President of Human Resources (HR).

**Fig. 1:**
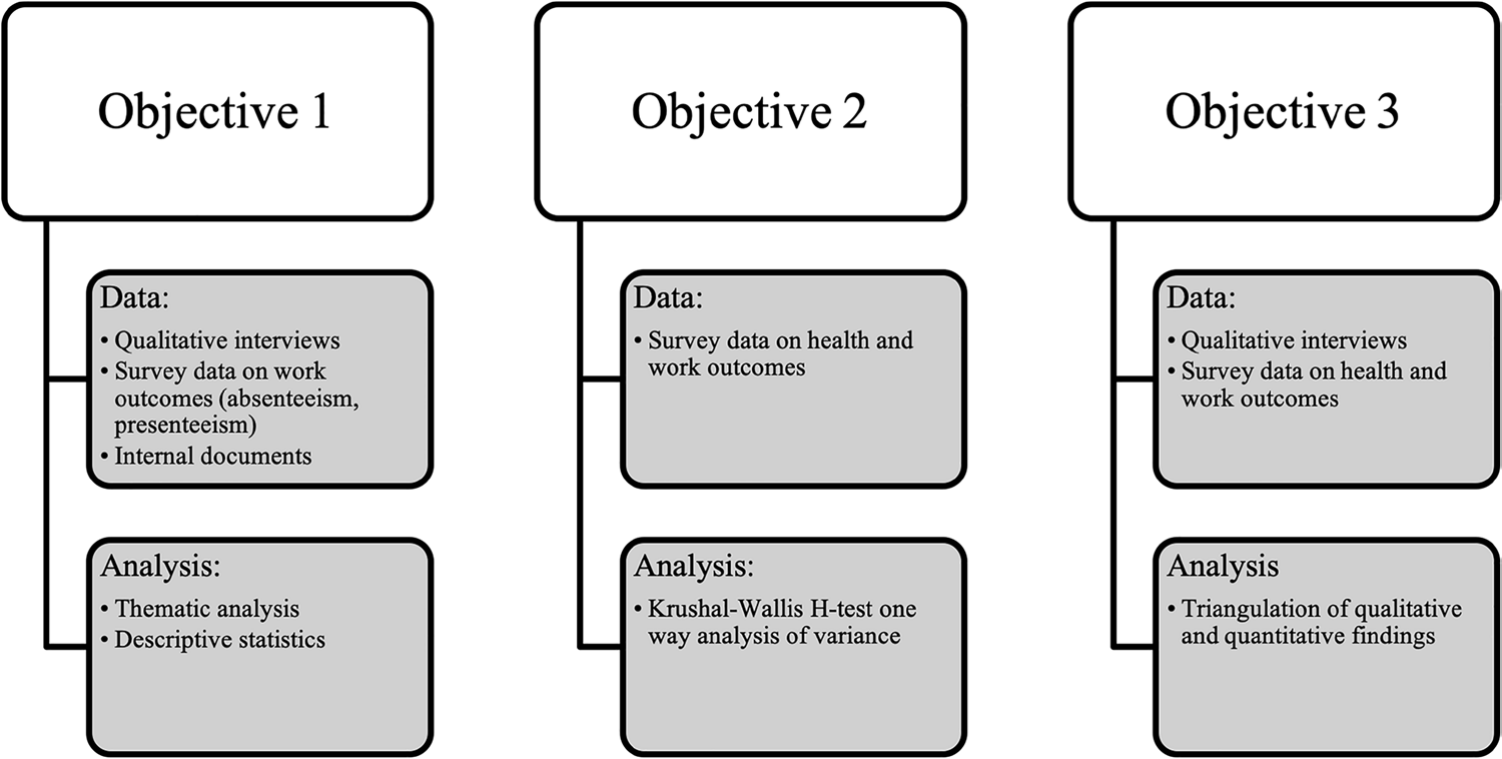
Methodological breakdown of the data and analysis involved with each of the three outlined objectives

Both qualitative and quantitative methods were used to address each of the objectives (Fig. 1). Starting from June 2020, data were collected concurrently from three main sources: (1) a workplace-wide sociodemographic survey; (2) qualitative interviews with self-identified carer-employees and key informants (e.g., managers and HR), and; (3) internal policies and procedures documents obtained from the organization’s HR department. The survey was open to participants for 4 weeks; during this time, interviews were being conducted.

### Recruitment

The link to the online survey and call for interview participants were distributed via e-mails from HR, which directed potential participants to the first author’s email. It was communicated that interview participants’ identities would be kept confidential by the researchers, and survey responses would be anonymous. In total, we interviewed 4 key informants and 5 carer-employees, recruited through the call for participants attached to the emails. From the surveys, we received 80 responses—43 full and 37 partial responses.

### Survey

The workplace wide survey was first circulated electronically at the end of June 2020, followed by three reminder emails. The survey was open to all employees in the workplace, with the incentive of winning a $100 Amazon gift card. Exclusion criteria consisted of employees who were contractors or consultants from other workplaces. The online sociodemographic survey contained questions related to employee age, gender, ethnicity, income, family status and self-identified carer status. As well, scales pertaining to COVID-19 effects on their work and family life, the status of workplace accommodations due to COVID-19, and general employee health and workplace culture were included. The online survey contained a consent page, outlining the purpose of the research and providing an opportunity to exit the survey at any time.

Health scales included were the SF-12 and CES-D-10, which are global measures of self-reported health and depression. The SF-12 contains two sub-components: the Physical Component Score (PCS) and Mental Component Score (MCS), which rates physical and mental health respectively (Maruish, 2012). The CES-D-10 is a short-form version of the validated Center of Epidemiological Studies scale, which assesses participant mood and depression risk in a population (Eaton et al., 2004). General self-efficacy (GSE-10) captured participants’ sense of self-belief surrounding coping ability (Chen et al, 2001). The Zarit burden scale was only presented to participants that indicated that they were carers, and assessed the extent of carer burden (Bédard et al., 2001). Work scales included were: job satisfaction (Rentsch & Steel, 1992), coworker support (O’Driscoll et al., 2004), work-family conflict and family work conflict (Netemeyer et al., 1996), schedule control (Thomas & Ganster, 1995), and family supportive supervisor behavior (Hammer et al., 2013). The World Health Organization’s Health and Work Performance Questionnaire (HPQ) scale was used to capture presenteeism and absenteeism, referring to loss of productivity while at work and unplanned missed work respectively (Kessler et al, 2003). All scales (Table 1) were selected for their reliability and validity.

**Table 1:** Scale range, scale direction, and interpretation of all survey-collected outcome variables

Scale	Number of items	Likert scale	Range	Scale direction
Self Reported Health (SF-12)	12	1–5	0–100	Higher score indicates better health; linearly transformed T scores based on 2009 US general population with a mean of 50 and standard deviation of 10; divided into mental health (MCS) and physical health (PCS) subscale
Center of Epidemiological Studies Depression CESD-10	10	0–3	0–30	Higher score indicates greater risk of clinical depression, scores of 11 or greater indicates high risk
General Self Efficacy (GSE)	10	1–4	10–40	Higher score indicates better self-confidence
Zarit Caregiver Burden (ZB)	12	0–4	0–48	Higher score indicates greater caregiver burden, score of 10–20 is considered mild burden, with scores > 20 considered high burden
Work Family Conflict (WFC)	5	1–5	5–25	Higher score indicates more conflict
Family Work Conflict (FWC)	5	1–5	5–25	Higher score indicates more conflict
Coworker Support (CWS)	4	1–6	4–24	Higher score indicates more sense of global (for both work and non-work reasons) support from colleagues
Absenteeism (HPQ)	1	1–10	NA	Higher score indicates more missed work, where 0 represents no lost work and negative scores represent overtime work
Presenteeism (HPQ)	1	1–10	0–100	Higher score indicates better productivity
Job Satisfaction (JS)	5	1–7	5–35	Higher score indicates increased satisfaction with paid work
Schedule Control (SC)	8	1–5	8–40	Higher score indicates greater freedom pertaining to work schedule
Family Supportive Supervisor Behaviour (FSSB)	4	1–5	4–20	Higher score indicates greater supervisor support of family conflicts

Other non-scalar survey questions included turnover intention, which was probed by asking if participants had considered leaving their job within the past 12 months, with the option of a “yes” or “no” response. COVID specific questions were selected after a thorough review and discussion with our research team. Given that one of our objectives of the environmental scan was to be able to understand how workplaces have changed with COVID-19 prior to implementation of a multi-level intervention, our selected questions were chosen to best reflect potential actionable changes for our future intervention. In our survey, we probe existing COVID accommodations, satisfaction with accommodations, care hours before and during COVID, and care work conflicts.

All survey data analysis was conducted on R. 4.0.3. Likert outcome variables for each participant were converted to numerical scores and summed according to scoring guidelines.

Descriptive or summary statistics were also generated for categorical data. For presenteeism, as the HPQ includes a prompt for retrospectively assessing presenteeism 12 months ago, a paired t-test was conducted to assess for significant differences in presenteeism at the time of the survey, and presenteeism 12 months ago. Two correlation matrices, a general matrix and one for carers, were developed using the Kendall method on pairwise data to assess collinearity using data collected from the surveys. The matrices also give insight to underlying relationships between outcome variables, helping explore objective 2 and suggest potential areas of improvement for objective 3. Data were tested for normality, linearity, homogeneity of variances, and outliers. Non-normal data were subjected to Levene’s test to assess heterkoskedacity. Multiple Kruskal Wallis-H tests was conducted to test differences in measured response variables between carers and non-carers, given the nonparametric nature of the data. While only 43 full survey responses were obtained, where possible, partial surveys were included for analysis if outcome variable scales were answered in full.

### Interviews

Interviews with key informant stakeholders (managers and HR) and carer-employees were conducted in order to gain a rich and nuanced understanding of workplace dynamic, culture, accommodations, and changes with COVID, as a supplement to quantitative data (Appendix A and B). From HR’s recruitment email, interested interview participants were directed to contact the first author to discuss eligibility and obtain informed consent. Eligibility requirements involved interview participants to be either: (1) currently or within the last 3 months an unpaid carer to a friend or family member; or 2) employed in a position of leadership or power over other employees (ex. manager, HR, team leads etc.…). Letters of information/consent were also provided to interview participants prior to the interview, and all participants verbally provided their informed consent. Survey participants were also prompted about the Canadian Standards Association’s Carer Standard and its suitability to their work environment. All interviews were completed over the phone, with verbal consent obtained from each participant. Interview prompts were semi-structured and, for the most part, the same between both stakeholders and carer-employees. Table 2 depicts the characteristics of each interview participant.

**Table 2:** Interview participant demographic information (N = 9)

Participant	Age	Sex	Job position	Inclusion criteria (Carer or Managers/HR)
Jaime	35–44 years	Female	Administrative	Carer
Christine	45–54 years	Female	Administrative staff	Carer
Kelly	55–65 years	Female	Technical staff	Carer
Kevin	35–44 years	Male	Technical staff	Carer
Anna	45–54 years	Female	Team lead	Carer
Kane	55–65 years	Male	Senior manager	Manager/HR
Dorothy	45–54 years	Female	HR personnel	Manager/HR
Cherie	45–54 years	Female	Manager	Manager/HR
Maria	45–54 years	Female	Manager	Manager/HR

With participant permission, interviews were audio recorded and transcribed verbatim after the surveys and analyzed thematically using NVivo 12. Participants were also invited to review transcripts and modify their responses after transcription. The full description of the process of thematic analysis is described in a prior publication (Ding &Williams, 2022). Pseudonyms are used herein to protect the anonymity of participants.

### Internal Documents

Several internal documents were provided to our research team from the organization’s HR, including information on organizational size, average wages, gender ratio, ethnicity and age distribution, as well as documents on benefits and accommodations, employment assistance program (EAP), and official policies on diversity, equal opportunity, and human rights. In addition to these, we also reviewed publicly available documents on the organization’s website, such as their health, safety, security and environmental performance documents. Research notes were also taken during meetings with HR to capture other contextual information that emerged in discussion.

### Rigour

Rigour was employed throughout all steps of the project, from conception to data analysis and reporting of findings. The mixed methods design was specifically selected in order to develop greater and nuanced understanding of the problem and results, as well as compensating for limitations of each method (Brown et al., 2015). Quantitative instruments were selected for their demonstrated reliability and internal validity across a number of contexts, and qualitative interview questions were developed with input from our research team and stakeholders from our partnered organization. Interview questions were also rehearsed with research team members several times during the design process for clarity. Research field notes, thick description, audio recording (with participant permission), and transcription was captured during all interviews to ensure credibility and transferability. The integration/triangulation of quantitative and qualitative data was achieved by first separately and sequentially analyzing the quantitative and qualitative data; followed by cross referencing each of the qualitative themes with a quantitative result. Critical reflexivity was practiced by maintaining a journal of researcher reflections during the design, data collection and analysis stages.

### Triangulation

Key findings related to our research questions were identified separately in quantitative and qualitative data, and then compared across the two types of data, consistent with a mixed methods analytic approach (O’Cathain et al., 2010). Findings were then triangulated using a modified version of Farmer et al.’s triangulation protocol (2006), where findings were classified into convergence, silence, and dissonance. Convergence refers to when both quantitative and qualitative data are in agreement on a specific theme or finding, whereas dissonance describes when quantitative and qualitative data differ. Silence refers to when a specific theme/finding is only present in either the quantitative or qualitative data. These findings were cataloged in a table format and presented in Appendix C.

## Results

### Workplace Context

The following section details the intricacies of the overall workplace, based on internal documents and meeting notes. The workplace investigated in this study is the Canadian division of a large international corporation in the engineering consultant field. The Canadian division is considered a large sized enterprise, employing approximately 4000 employees prior to COVID-19. This workplace specialized in oil and gas, with multiple offices located in all Canadian provinces, although at the time of data collection, majority of the employees were working from home.

Overall, employees tend to be highly educated and technically skilled, with a mean hourly wage (benefits not included) of $43.01, with a standard deviation of $21.01. Benefits comprise of approximately 20% of base wage and include an EAP, health and dental care, and insurance. As a consulting labour force, the majority of the technical workforce charges clients per hour for their time and expertise, which is augmented with smaller overhead staff; this, therefore, leads to large variations in wages. Fieldwork or labwork tend to be more common responsibilities for junior or newer employees, with senior employees taking on more bureaucratic responsibilities. A diverse range of ethnic backgrounds are represented within the workplace. Notably, the majority of employees identify as male, with approximately one third of the workforce identifying as female. The mean age of the workforce is 45.3 years, with a standard deviation of 13.4 years.

The workplace has a number of initiatives engendering a healthy workforce, with many publicly viewable and easily accessible documents and statements regarding the organization’s stance on workplace health, safety and security. In addition to insurance-provided dental and health benefits, the Employee Assistance Program (EAP) is fairly robust, with resources such as counseling, training, webinars, and phone lines across many topics including, but not limited to, depression, anxiety, family issues, and caregiving. Interestingly, this workplace also operates several internal support networks, including one for caregiving specifically. These initiatives are all introduced during employee orientation and onboarding for new employees; however, they are not regularly reinforced or publicized, outside of the occasional email newsletter. As an engineering firm, physical occupational health is heavily addressed during meetings and email alerts, while initiatives for mental health or work-life balance are not as frequently discussed.

### Descriptive Data

While the survey sample consisted of 80 responses, only 43 were full responses, although another additional 6 responses were mostly (over 90% completion) full responses. These incomplete responses were used, where possible, for summary statistics on each specific question, but excluded during pairwise analysis. Of the 80 respondents, approximately 13 identified as carers and 14 identified as team leaders (in a position with authority over other employees). Carer and team leader designation were not mutually exclusive, with some team leaders also identifying as carers. Due to the nature of the nature of recruitment, it is unlikely that employee respondents were direct reports to the participating supervisors. The interview sample (N = 9) were also potentially respondents to the survey, although we did not confirm their participation in the survey to maintain anonymity of responses. For the remainder of this paper, we shall focus on the experiences of carers compared to non-carers; however, we recognize that there are a wide variety of experiences within other groups, such as parents with young children or team leaders.

Table 3 outlines the basic sociodemographic profile of respondents. Overall, participants in both the surveys and interviews were located across Canada, most commonly in full-time employment and married; the age distribution of the participants was fairly even, with the 25–34 age group most represented (see Table 3). Participants were university educated, mid to senior level employees, and the majority had salaries over $50,000 annually. Survey respondents appear to skew slightly younger than the workplace baseline as described in the previous section, although our survey respondents also tended to be high-income earners and roughly followed the same gender distribution of the overall workplace.

**Table 3:** Descriptive characteristics of survey respondents (N = 49)

Demographic variable	Non-carer (n = 36) Count (Frequency)	Carer (n = 13) Count (Frequency)	Overall (N = 49) Count (Frequency)
Gender			
Female	12 (33.3%)	5 (38.5%)	17 (34.7%)
Male	19 (52.8%)	5 (38.5%)	24 (49.0%)
Prefer not to say	1 (2.8%)	0 (0%)	1 (2.0%)
Missing	4 (11.1%)	3 (23.1%)	7 (14.3%)
Age			
18–24 years	3 (8.3%)	0 (0%)	3 (6.1%)
25–34 years	11 (30.6%)	1 (7.7%)	12 (24.5%)
35–44 years	9 (25.0%)	1 (7.7%)	10 (20.4%)
45–54 years	3 (8.3%)	4 (30.8%)	7 (14.3%)
55–64 years	6 (16.7%)	3 (23.1%)	9 (18.4%)
65 + years	0 (0%)	1 (7.7%)	1 (2.0%)
Missing	4 (11.1%)	3 (23.1%)	7 (14.3%)
Marital			
Common-law	2 (5.6%)	0 (0%)	2 (4.1%)
Married	23 (63.9%)	9 (69.2%)	32 (65.3%)
Single	7 (19.4%)	0 (0%)	7 (14.3%)
Other	0 (0%)	1 (7.7%)	1 (2.0%)
Missing	4 (11.1%)	3 (23.1%)	7 (14.3%)
Race			
Latin American/Hispanic	1 (2.8%)	1 (7.7%)	2 (4.1%)
Prefer not to say	3 (8.3%)	0 (0%)	3 (6.1%)
South Asian	2 (5.6%)	0 (0%)	2 (4.1%)
Southeast Asian	1 (2.8%)	0 (0%)	1 (2.0%)
West Asian	1 (2.8%)	0 (0%)	1 (2.0%)
White	23 (63.9%)	8 (61.5%)	31 (63.3%)
Arab	0 (0%)	1 (7.7%)	1 (2.0%)
Missing	5 (13.9%)	3 (23.1%)	8 (16.3%)
Employment contract type			
Contract full-time	1 (2.8%)	1 (7.7%)	2 (4.1%)
Full-time	27 (75.0%)	8 (61.5%)	35 (71.4%)
Part-time	3 (8.3%)	1 (7.7%)	4 (8.2%)
Seasonal full-time	1 (2.8%)	0 (0%)	1 (2.0%)
Missing	4 (11.1%)	3 (23.1%)	7 (14.3%)
Education			
High school diploma/GED	1 (2.8%)	1 (7.7%)	2 (4.1%)
College/apprenticeship/technical diploma or equivalent	4 (11.1%)	2 (15.4%)	6 (12.2%)
Master’s or equivalent	10 (27.8%)	1 (7.7%)	11 (22.4%)
Doctoral or equivalent	1 (2.8%)	0 (0%)	1 (2.0%)
Missing	4 (11.1%)	3 (23.1%)	7 (14.3%)
Income			
$30,000–$49,999	1 (2.8%)	1 (7.7%)	2 (4.1%)
$50,000–$69,999	8 (22.2%)	3 (23.1%)	11 (22.4%)
$70,000–$99,999	9 (25.0%)	1 (7.7%)	10 (20.4%)
Over $100,000	11 (30.6%)	3 (23.1%)	14 (28.6%)
Prefer not to answer	3 (8.3%)	2 (15.4%)	5 (10.2%)
Missing	4 (11.1%)	3 (23.1%)	7 (14.3%)
Tenure			
Under 1 year	7 (19.4%)	0 (0%)	7 (14.3%)
1–4 years	5 (13.9%)	1 (7.7%)	6 (12.2%)
10–14 years	6 (16.7%)	5 (38.5%)	11 (22.4%)
14–19 years	3 (8.3%)	2 (15.4%)	5 (10.2%)
5–9 years	9 (25.0%)	1 (7.7%)	10 (20.4%)
Over 20 years	2 (5.6%)	1 (7.7%)	3 (6.1%)
Missing	4 (11.1%)	3 (23.1%)	7 (14.3%)
Supervisor			
No	24 (66.7%)	12 (92.3%)	36 (73.5%)
Yes	12 (33.3%)	1 (7.7%)	13 (26.5%)

### *Work Experience of Survey Respondents (N* = *49)*

Presenteeism and absenteeism were evaluated using the HPQ scale using measures of absolute presenteeism (ratio of one’s actual work performance with one’s potential performance) and absolute absenteeism (number of work hours lost) over the past 4 week period. Absolute presenteeism and absolute absenteeism appear to be high across the entire sample (including both carers and non-carers). A mean absolute presenteeism score was calculated at 79.8, and a mean absenteeism score of 12.1 was obtained; this indicates that employees were, on average, performing at 79.8% of their full potential, and that approximately 12.1 work hours were lost per employee over the 4 week period. A paired sample t-test found that employees had significantly higher presenteeism during the current year (M = 79.8, SD = 11.7) when compared to the previous year (M = 86.6, SD = 7.13), indicating productivity losses during COVID, t(43) = 4.97, p < 0.001. Other work related outcomes can be viewed in Table 4.

**Table 4:** Mean scores, median, min, max, and missingness of the work and health related variables captured in the survey (N = 49)

Outcome Variable	Non-Carer	Carer	Overall
	(n = 36)	(n = 13)	(N = 49)
Self reported health physical component (PCS)			
Mean (SD)	55.3 (4.58)	56.5 (4.63)	55.59 (4.56)
Median [Min, Max]	56.7 [43.3, 62.4]	56.5 [48.1, 64.47]	56.71 [43.30, 64.47]
Missing	1 (2.8%)	1 (7.7%)	2 (4.1%)
Self reported health mental component (MCS)			
Mean (SD)	48.5 (10.0)	46.7 (11.6)	48.07 (10.37)
Median [Min, Max]	50.1 [22.1, 62.4]	48.4 [21.5,60.2]	50.08 [21.52, 62.38]
Missing	1 (2.8%)	1 (7.7%)	2 (4.1%)
Depression (CESD-10)			
Mean (SD)	6.74 (5.82)	7.50 (7.59)	6.93 (6.24)
Median [Min, Max]	5.00 [0, 21.0]	5.00 [0, 24.0]	5.00 [0, 24.0]
Missing	2 (5.6%)	1 (7.7%)	3 (6.1%)
General self-efficacy (GSE)			
Mean (SD)	32.4 (3.76)	31.8 (3.84)	32.2 (3.75)
Median [Min, Max]	32.0 [24.0, 40.0]	31.0 [27.0, 38.0]	32.0 [24.0, 40.0]
Missing	2 (5.6%)	1 (7.7%)	3 (6.1%)
Zarit burden (ZB)			
Mean (SD)	NA (NA)	20.1 (10.5)	20.1 (10.5)
Median [Min, Max]	NA [NA, NA]	16.5 [6.00, 41.0]	16.5 [6.00, 41.0]
Missing	36 (100%)	1 (7.7%)	37 (75.5%)
Turnover			
N/A	4 (11.1%)	2 (15.4%)	6 (12.2%)
No	19 (52.8%)	6 (46.2%)	25 (51.0%)
Yes	13 (36.1%)	5 (38.5%)	18 (36.7%)
Work-family conflict (WFC)			
Mean (SD)	13.7 (5.03)	14.6 (6.33)	13.9 (5.30)
Median [Min, Max]	15.0 [5.00, 22.0]	13.5 [5.00, 25.0]	15.0 [5.00, 25.0]
Missing	4 (11.1%)	3 (23.1%)	7 (14.3%)
Family work conflict (FWC)			
Mean (SD)	10.4 (4.23)	11.7 (4.60)	10.7 (4.30)
Median [Min, Max]	10.0 [5.00, 24.0]	11.5 [6.00, 22.0]	10.0 [5.00, 24.0]
Missing	4 (11.1%)	3 (23.1%)	7 (14.3%)
Coworker support (CWS)			
Mean (SD)	14.9 (4.55)	9.30 (3.09)	13.5 (4.85)
Median [Min, Max]	16.0 [5.00, 22.0]	9.50 [4.00, 15.0]	14.5 [4.00, 22.0]
Missing	4 (11.1%)	3 (23.1%)	7 (14.3%)
Missing	4 (11.1%)	2 (15.4%)	6 (12.2%)
Absolute presenteeism (HPQ)			
Mean (SD)	78.2 (12.6)	84.5 (6.88)	79.8 (11.7)
Median [Min, Max]	80.0 [50.0, 100]	90.0 [70.0, 90.0]	80.0 [50.0, 100]
Missing	3 (8.3%)	2 (15.4%)	5 (10.2%)
Absolute presenteeism previous year (HPQ)			
Mean (SD)	85.5 (7.11)	90 (6.32)	86.6 [7.13]
Median [Min, Max]	90 [70,100]	90 [80,100]	90 [70,100]
Missing	3 (8.3%)	2 (15.4%)	5 (10.2%)
Job satisfaction (JS)			
Mean (SD)	26.0 (5.53)	27.2 (3.40)	26.3 (5.07)
Median [Min, Max]	27.0 [14.0, 34.0]	26.0 [22.0, 32.0]	27.0 [14.0, 34.0]
Missing	3 (8.3%)	2 (15.4%)	5 (10.2%)
Schedule control (SC)			
Mean (SD)	30.1 (6.31)	30.5 (6.65)	30.2 (6.32)
Median [Min, Max]	31.0 [17.0, 40.0]	32.0 [21.0, 40.0]	31.0 [17.0, 40.0]
Missing	3 (8.3%)	2 (15.4%)	5 (10.2%)
Family supportive supervisor behavior (FSSB)			
Mean (SD)	14.1 (4.39)	14.4 (4.12)	14.2 (4.28)
Median [Min, Max]	14.0 [4.00, 20.0]	14.5 [7.00, 20.0]	14.0 [4.00, 20.0]
Missing	4 (11.1%)	3 (23.1%)	7 (14.3%)

**Fig. 2:**
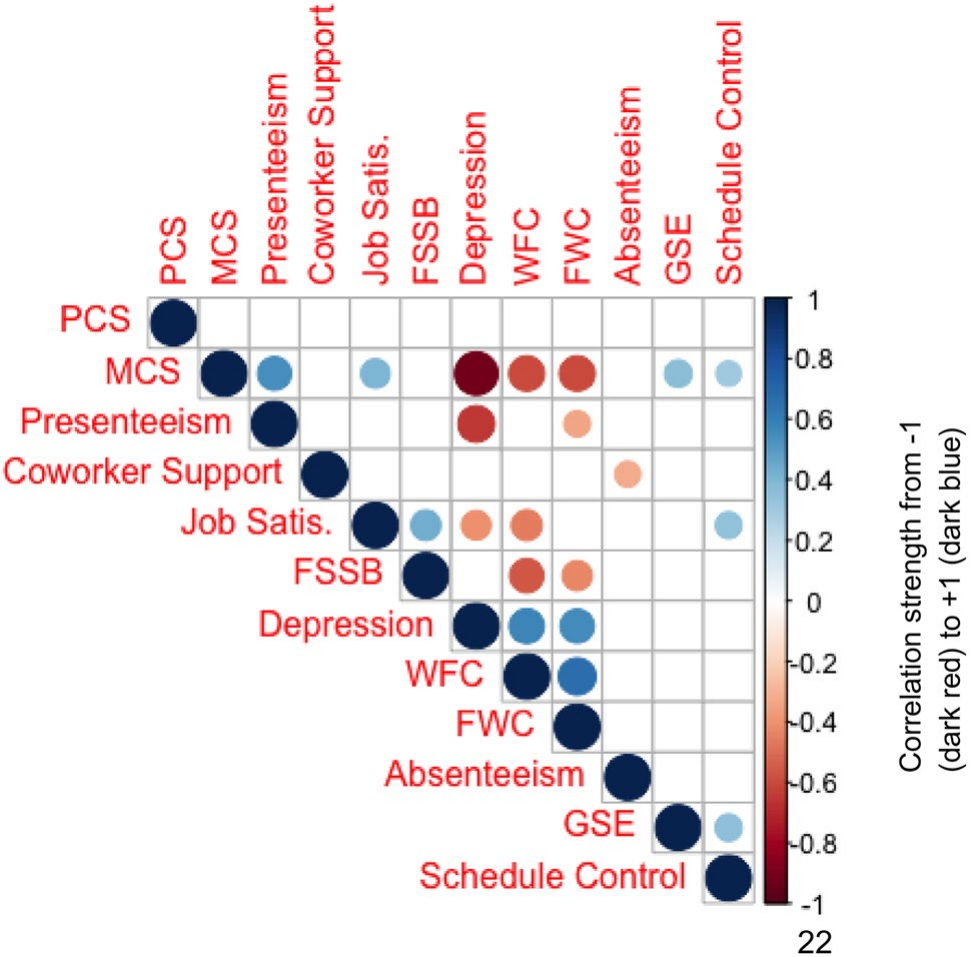
Correlation matrix plot across entire sample, where only significant (p < 0.05) associations are reported

**Fig. 3:**
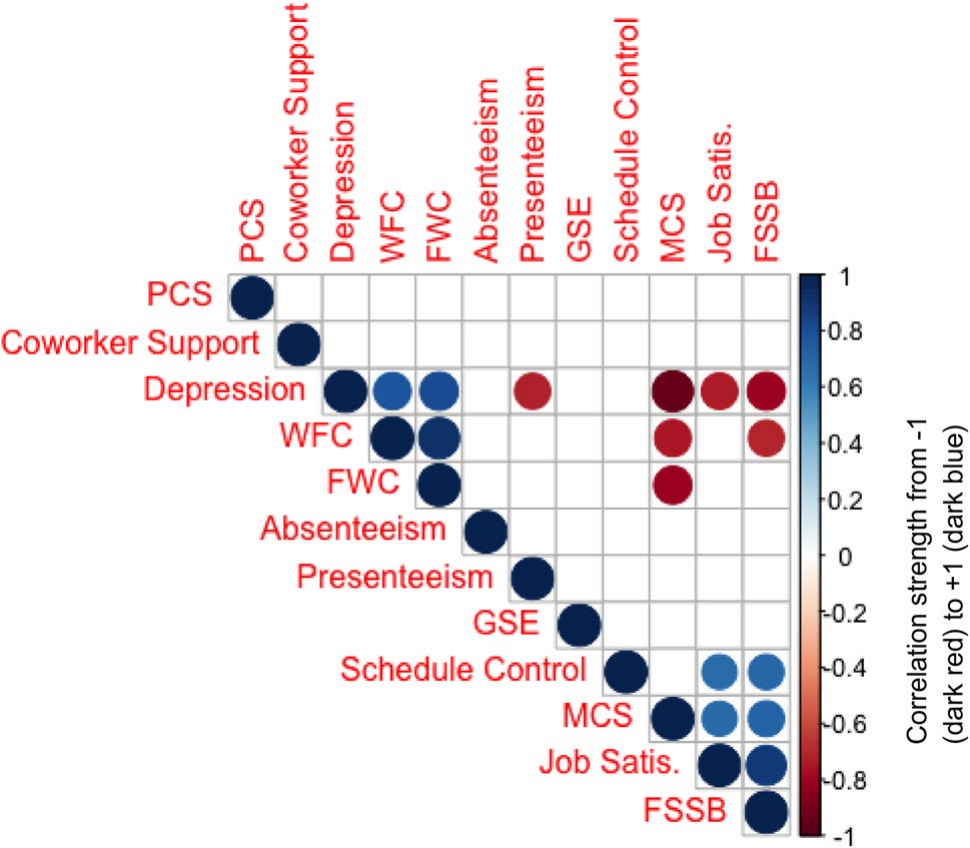
Correlation matrix plot for carer-employee responses, where only significant (p < 0.05) associations are reported

The correlation matrices, Fig. 2 and Fig. 3, display several significant associations between health and work variables, suggesting weak-moderate associations. Overall, associations were more prevalent and stronger among the carer cohort, although this may be a function of the smaller sample size. Across the entire sample, we observe several notable correlations of interest. The mental health component of the SF-12 (MCS) was strongly and negatively associated with depression (CESD-10), work-family conflict (WFC), and family-work conflict (FWC), while being positively correlated with presenteeism scores (HPQ), general self-efficacy (GSE), and schedule control (SC). Presenteeism scores were strongly and negatively associated with depression risk. Work-family conflict, family-work conflict, and depression appear to be correlated with each other, while family supportive supervisor behaviour (FSSB) is negatively correlated with work-family and family-work conflict.

Qualitative data was used to capture the nuances of workplace culture from both the perspectives of carers and stakeholders, in order to compliment descriptive findings pertaining to objective 1 and 2. It was observed that the current workplace culture was characterized as: high pressure, having a lack of work life balance and a large variation in managerial attitudes towards caregiving responsibilities.

Due to the nature of the workplace as a consulting firm, there is high pressure to meet client demands, producing high work burden and stress. One participant remarked “we’re a consulting business. So there’s this constant pressure that they’re billable to clients all the time. That does cause people pressure and stress”- Dorothy.

This pressure has compounded due to COVID-19, due to a large proportion of the workforce working from home and generating unique stresses there as well. Another participant described the pressure to simultaneously know how to use unfamiliar technology while maintaining their billability, “They haven’t provided formal training to use [virtual conferencing technology]…I don’t know how people are just supposed to morph and just know how to do this stuff….how much time should you spend playing around with the technology? If they also still want us to be as utilizable as possible, that’s meaning, as much of our time as possible to be billed to clients, not just overhead training. So how much am I supposed to be doing that on my own time? Am I supposed to do that on work time?” -Kelly.

This pressure also generates unhealthy work-life balance with pressure to prioritize work over personal life. One participant indicated that this pressure had increased since lockdown measures with COVID-19:

You just have to suck it up. I’m only too happy to have a job. When the lockdown happened, there wasn’t a lot of work to do. We had to send staff on layoffs, and some people just quit. And basically they were just [a few of us] in one department. And then the workload just became too much for us to handle. It’s crazy. It’s horrible. -Anna.

Most notably, direct managerial attitudes were identified as being most conducive to establishing and maintaining work culture prior to and during COVID measures. One carer-employee participant described their experience with an unsupportive direct manager prior to COVID-19, “I didn’t feel like there was a support system behind me, or I didn’t know who I could reach out and talk to. I really felt I had no power. And that if I had said, I would like to take two weeks off because I’d like to spend that time with my [parent], I don’t feel like the support would have been there. And I feel like my job would have been at risk. -Jaime”.

However, positive attitudes towards family responsibilities not only improve employee satisfaction but also employee retention. Another participant describes the benefit of being given flexible work arrangements prior to COVID-19, “The flexibility was huge; that made my quality of life. If I hadn’t had a workplace that would allow me to work from home, it would have been devastating. They got me for life by letting me do this. They got my loyalty, I’ll be always grateful for that. Because it allowed me to be able to be there for my [parent]…And if they never gave me another cost of living increase ever again, I wouldn’t really feel entitled to complain. Because how do you put a price tag on that? Like, how many tens of thousands of dollars is it worth teams to do that? Well? A lot, right.” -Kelly.

Overall, given the unique sector described by our collected data, it is apparent that even prior to COVID-19, this particular labour force experienced high stress and high workload, with large variation in managerial attitudes towards family responsibilities such as caregiving.

### Employee Health

Despite the ongoing pandemic at the time, employees generally report good health, as indicated by the SF-12 and CES-D scales in Table 4, with the distribution right skewed. However, approximately a third of the survey sample had CES-D scores over 10, indicating a high clinical risk of depression. General self-efficacy (GSE) across the survey sample had a mean score of 32.2, indicating fairly high levels of self-efficacy.

Many participants spoke about the efforts that their HR department was taking in order to promote mental health resilience during COVID-19. One manager remarked “we get them in place, and resources to make sure that we’re, we’re spending that quality time with our people. And we’re checking in really often and again, but I don’t think we do it enough. And so I think that needs to happen a little bit more. And we need to really get a really good sense of are people truly doing well? Are they really? Are they really mentally focused? And is this something that works for them or not? So I think we need to get a little bit better with that and be really, really looking, zoning in on workloads and whether or not they’re manageable.” -Cherie.

Another manager describes the deleterious effects of employees with unsupported mental health due to burnout, “they’re not able to stay efficient, or work because their attentions are distracted. But they also feel they can’t stop working because they can’t afford not to stop working. And so, you know, that just causes the stress cycle, right?” -Kane.

### COVID-19 Specific Workplace Changes

**Fig. 4:**
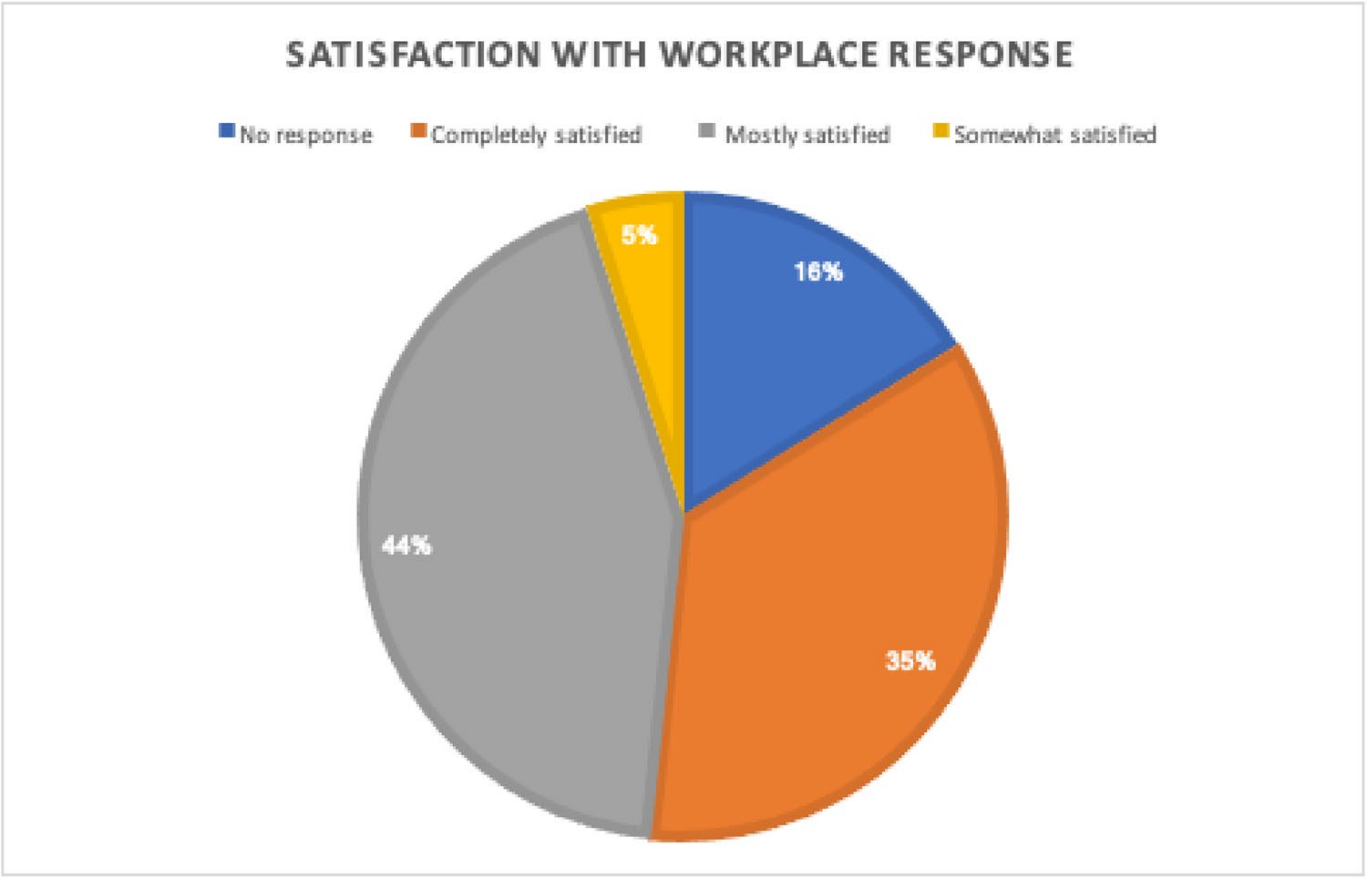
Employee satisfaction with their employer’s response to COVID-19 (N = 66)

Since the Spring of 2020, a large component of the workforce has been able to transition to work-from-home. However, given the nature of the industry, there are many employees still required to work onsite or in the field. The workplace has also laid off several hundred employees due to COVID-19 and the related project cancellations. Generally, most respondents were satisfied with the workplace’s adaptations to COVID (Fig. 4).

**Fig. 5:**
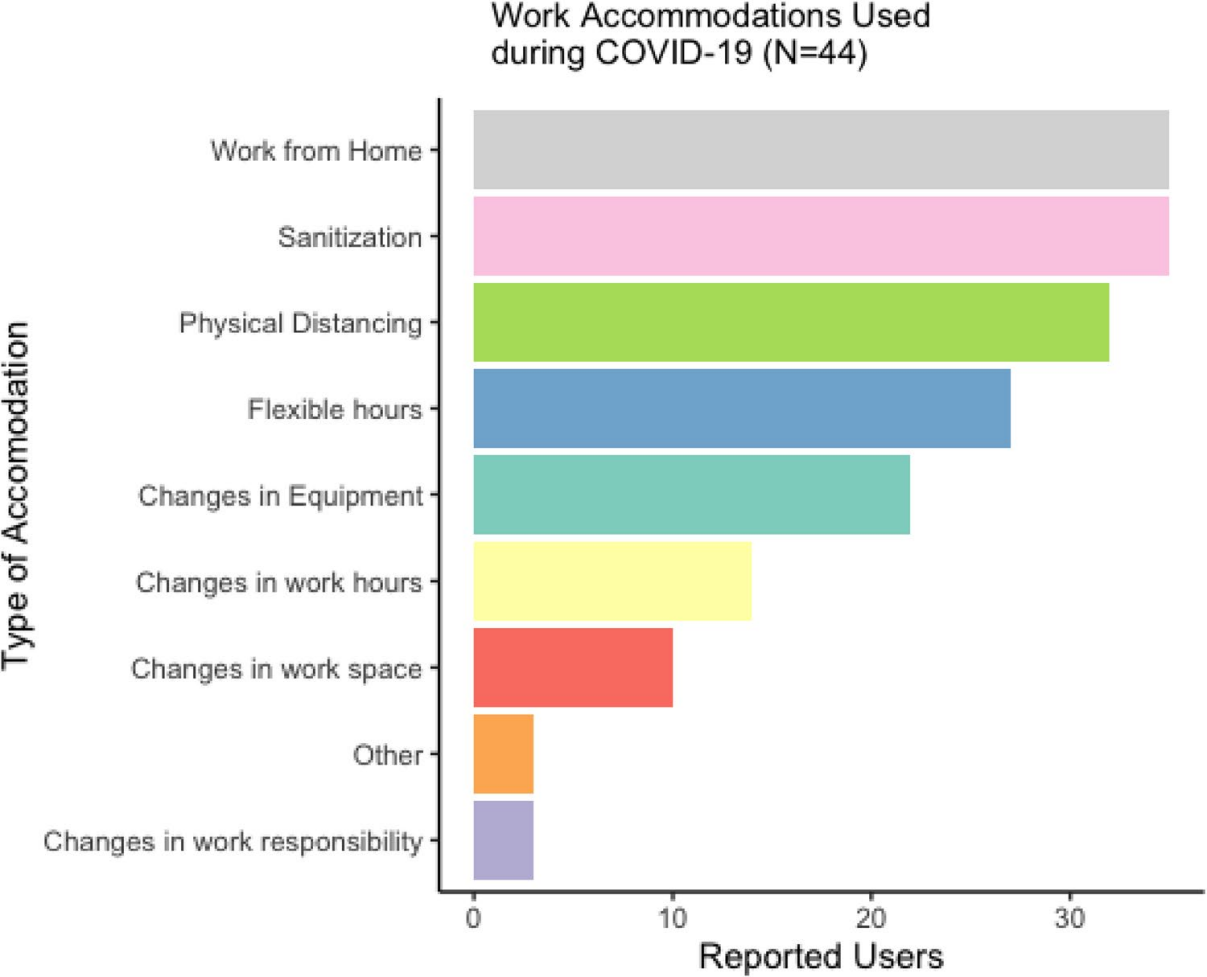
Employee- reported COVID-19 accommodations (N = 44)

The most common work accommodations used were work from home arrangements and sanitization of work spaces (Fig. 5). Approximately 35% of respondents (N = 22) indicated that they were completely satisfied with the company’s response to COVID, and another 44% (N = 29) indicated that they were mostly satisfied. When prompted about post-COVID accommodations, the majority indicated that they preferred to keep flexible work arrangements, such as work from home, as permanent.

When probed about whether flexible work options would be continued to be offered post-COVID, one manager stated: “100%, yes. I would say reluctancy was much more on the employee side to work from home [prior to COVID]. But I think the pendulum has definitely swung to where the employees want to work from home more. And it’s now up to [management], where we are going to take advantage of the fact that we can implement a much more robust work-sharing, work from home, partial office partial working from home situation for many more people that I think will embrace and take advantage of the opportunity” -Kane.

Communication during COVID has notably been altered, with some participants describing communication as strictly professional. While some participants state that it is beneficial due to less wasted time, others describe a loss of community. “we haven’t had a chance to build up that sense of community. Maybe because we’re each in our busy little spot “ -Christine.

Another employee described the technical challenges of collaborating virtually on projects, “sometimes, like one hand doesn’t know what the other one is doing. There’s too many project managers and they’re not communicating among the project managers; staff get caught in the middle, and they’re just pulled in too many directions. With everyone working from home that’s become definitely more prevalent there. When everyone was working in the office, we’d have an in person meeting and then everyone would on a weekly basis, we’d all say, Okay, this is what I’m up to. I’m going to be going here next Tuesday. And then someone was like, Oh, well, what about this? There was better communication and project coordination [prior to COVID]”.-Kevin.

In general, there are large variations in changes to workload with COVID. Many participants describe the workload as constant or having increased. One employee in a senior role stated, “the workload has increased because people are laid off..[it’s] technically much higher during this COVID-19, because you have all these projects with fewer people to do it. I can say I’m doing the job of three people combined.” -Anna.

Regardless, all employees touted the benefit and desire to remain working from home.

### Carer-Employees

**Fig. 6:**
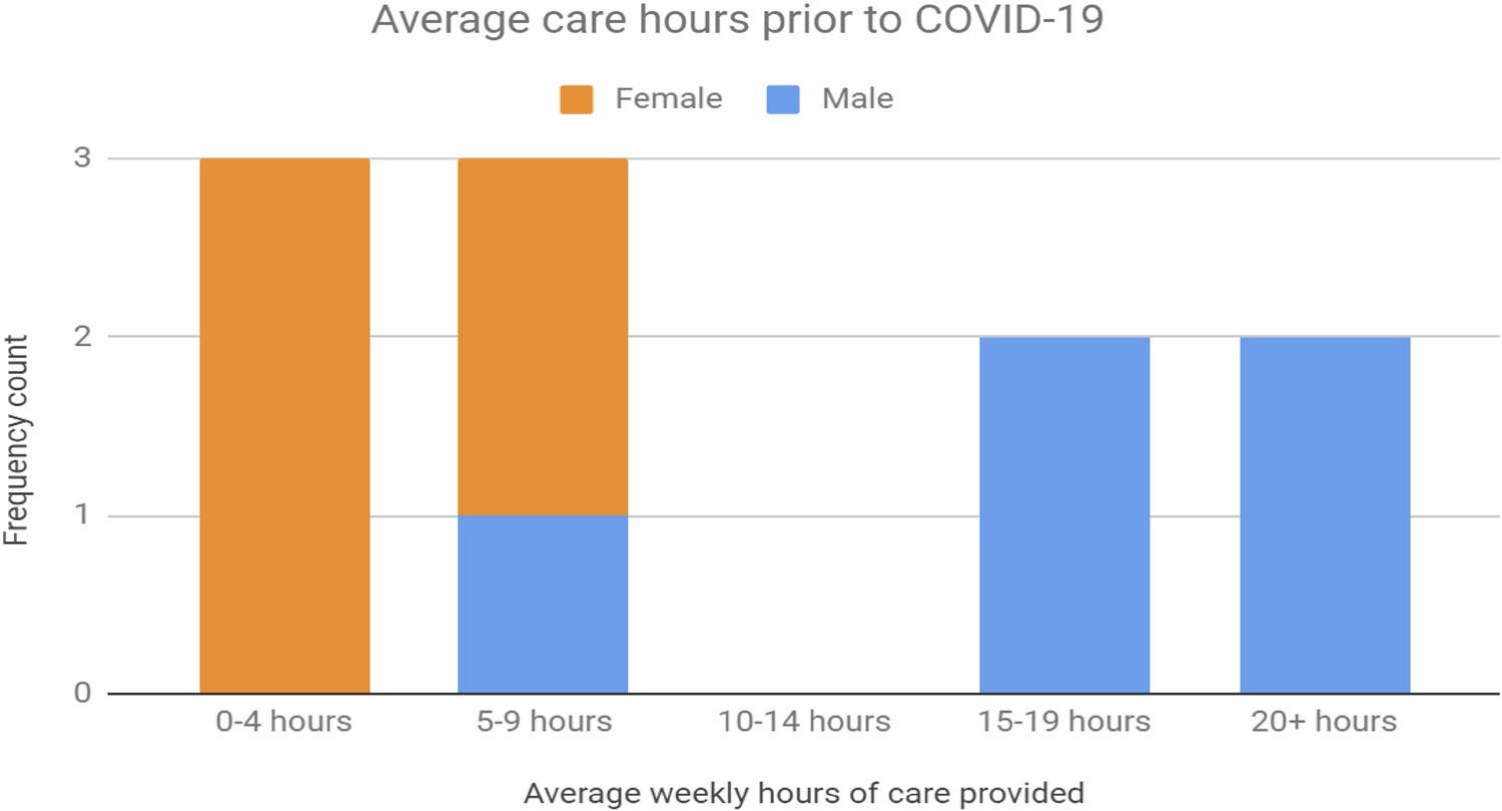
Average weekly hours of care prior to COVID-19

**Fig. 7:**
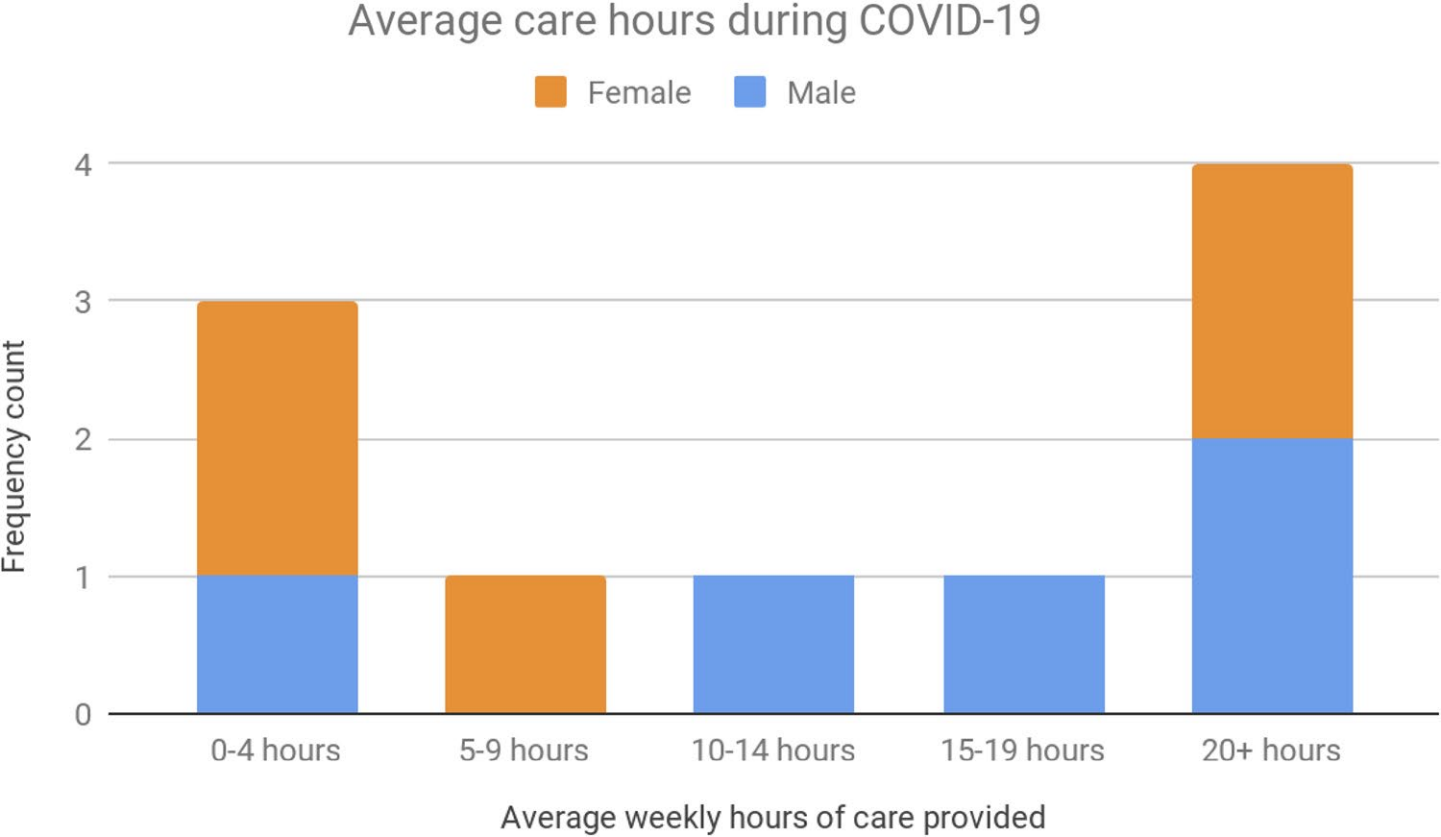
Average weekly hours of care during COVID-19

Of the 13 carer-employees in the sample, weekly care hours prior to COVID-19 followed a bi-modal distribution with clusters around the 0–9 h (N = 6) and the 15–20 + hours (N = 4) (Fig. 6). However, after the onset of COVID-19, the distribution sharpened toward more hours, with 5 carer-employees now indicating that they were providing over 15–20 + hours of weekly care (Fig. 7). There were an equal number of male (N = 5) and female carer-employees (N = 5), with 3 declining to identify their sex.

Table 4 depicts carer burden as measured by the Zarit Burden Index score. A mean score of 20.1 was obtained among the sample of 13 carers. Half of the carer sample had scores greater than 17, indicating high care burden.

The results of multiple individual Kruskal–Wallis tests between carers and non-carers and the selected response variable were compiled and presented in Table 5. The only observed significant difference in response variables between the two groups is in rated coworker support, with carers indicating lower perceived coworker support. Otherwise, carers do not differentiate from non-carers in any other outcome variable.

**Table 5:** Compiled Kruskal–Wallis one way analysis of variance tests between carer and non-carer groups

Response variable	df	H-value	p-value
Self-reported health (PCS)	1	0.22646	0.6342
Self-reported health (MCS)	1	0.078757	0.779
General self-efficacy	1	0.43484	0.5096
Depression	1	1.1504	0.2835
Absenteeism	1	0.11477	0.7348
Presenteeism	1	0.48426	0.4865
Job satisfaction	1	0.57986	0.4464
Schedule control	1	2.2313	0.1352
Work-family conflict	1	0.079201	0.7784
Family-work conflict	1	2.4997	0.1139
Family supportive supervisor behavior	1	0.007936	0.929
Co-worker support	1	5.621	0.01775***

**Fig. 8:**
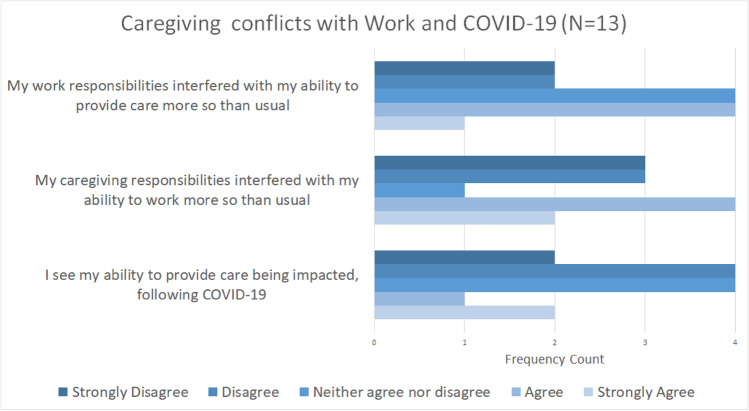
Carer self-assessment of the direction of work-care and care work conflicts during COVID-19

Carers in general report varying levels of conflicts between work, caregiving, and their personal time as seen in Fig. 8. Work responsibilities interfered with caregiving, but caregiving responsibilities were more likely to interfere with work.

Carers identified several conflicts, such as: unsupportive work environment, lack of knowledge on existing policies, and changes in caregiving due to COVID-19.

Unsupportive work environments generally fostered conflicts between work and caregiving, which resulted in paid work taking precedence over caregiving. As one participant described:

“I feel like if I take time off work, I risk being deemed like I’m not working as much as I should be—I feel guilty…When my [parent] got diagnosed with cancer, [they] were given less than three months to live. And I worked every day…I felt like I couldn’t take time off work to go be with [them] …you’re feeling guilty and there’s still work expectations. And I’m not wanting to cheat out on work by any means. But there’s things I look back on, and I’m like, why did I put work first?”- Jaime.

Carers in general were not aware of formal existing policies and resources within the workplace and preferred use of informal arrangements discussed with their direct supervisor. Alternatively, carers used vacation/sick days or part-time off (PTO) which allowed them to manage caregiving without involvement of other supervisors or employees. As previously identified, the lack of formal use of policies/procedures may relate to unsupportive work culture and managerial attitudes. One carer identified the challenge in trying to navigate and locate information on a specific benefit on the internal website, “I don’t know, I couldn’t find a document. I looked on the websites, I searched to find for [key word]. Nothing.” -Kelly.

This is incongruent with what in actuality is offered by the workplace. Supervisors, while having varying knowledge of carer resources, on average identified more resources than what carers were aware of or utilized, including: employee assistance programs (EAPs) specifically for caregiving, an internal carers network, formal telecommuting arrangements, professional development check-ins, webinars, condensed hours and other benefits.

Other challenges were caregiving conflicts introduced by COVID-19. With provincial restrictions set on household visits, physical distancing, and long-term care, many carers identified that the ways that they provide care had changed, with effects on their personal time and work life. Some carers indicated that caregiving is now a more singular process given that outside help is not possible due to COVID restrictions. Kelly explains, “before COVID, my brother would come over for three or four hours, most weekends, he’d spell me off for an afternoon or anything and that would allow me time one on one with my partner or to do errands”. Others describe how community level services have been reduced, leading to greater onus for carers to provide these services themselves, “I also was expecting handi-dart [to] pick her up and take her to the senior center so she can be busy there. So that cannot be done so it’s basically just the activities that we do together here with her, only myself and her [now].” -Christine.

Another participant explained their reservations about a return to the physical workplace due to ramifications on their family members’ health and safety via potential exposure, “Do I want to risk my [care recipient] from someone that I’ve maybe made contact with? We’re supposed to be encouraged to go into the office from time to time and [I] take transit. I have made the office aware that I don’t really want to take transit because I could have the opportunity to see my [care recipient] coming up. But, how do you manage that going forward? If I can never see [them] if I’m taking transit, and I eventually have to go back to work and take transit, what happens?” -Jaime.

This same participant describes changes in levels of concerns as they are unable to see their grandparent in the nursing home, “You can’t see [them]…you know, [they] fell last week and didn’t even tell us, the care home told us…[care recipient] doesn’t tell me what the schedule is and [they] never call me back. So it’s really the onus is on me to get a hold of [them]”. -Jaime.

These additional barriers to caregiving were noted to cause increased stress and anxiety to some carers. However, for other carers the extra time due to work from home arrangements has allowed more personal time, thereby mitigating some of the time constraints.

The HR department noted that they are interested in enacting changes to their workplace to foster a more supportive and carer-friendly work environment due to concerns in the past. One supervisor commented that, “[supporting carers is] vitally important. We come to work to support our families and live a life. Not come to work because it’s your life …we all have to deal with it at some point. You know, we as a company, owe it to our employees to support them through those hard times.” -Kane.

## Discussion

The purpose of this environmental scan is to generate contextual knowledge regarding baseline assessments of workplace culture, health, satisfaction, and changes to a large sized Canadian firm during the COVID-19 pandemic. This was done in order to facilitate the implementation of a tailored carer-supportive workplace policy intervention and ensure that such an intervention was grounded in the local context of the workplace. To our knowledge, this is the first study of its kind, utilizing a mixed-methods framework in evaluating the workplace context, with a focus on carer outcomes, during COVID-19.

We set out to answer the following questions:

- OBJECTIVE 1: In what ways has the paid work experience changed with COVID-19?
- OBJECTIVE 2: Do the work and health outcomes of carer-employees differ significantly from non-carer/regular employees?
- OBJECTIVE 3: How can workplaces adapt to support their employees, caregiving or otherwise?

### OBJECTIVE 1: In What Ways has the Paid Work Experience Changed with COVID-19?

To address our first objective, it is necessary to establish the work environment prior to COVID. Our qualitative findings found the nature of the workplace was characterized as a high stress environment, prior to and during COVID. This was reflected in an emphasis on achieving client deliverables and bill-ability that signals the prioritization of productivity within the workplace. Interview themes specific to working from home during the pandemic speak to: higher overhead costs and administrative work, more technological challenges, as well as the pervasive culture of bill-ability. Quantitative data was silent in this area.

We found convergent qualitative and quantitative evidence that the paid work experience has been changed for both carers and non-carers due to COVID. Qualitative themes describe the transition to remote working, with burdens such as a decline in interpersonal work relationships and communication issues. Complimenting this, survey results showed convergent evidence that, during the pandemic, work outcomes such as presenteeism increased when compared to previous years. Participants reported a mean presenteeism score of 86.59 in the previous year (2019), compared to presenteeism of 79.78 during June 2020; representing a significant increase in presenteeism (drop in productivity). Mean absenteeism was reported at approximately 12.1 h of work lost in the past month (June 2020). Absenteeism scores only cover the prior 4 week period and, as such, disallow comparability prior to the pandemic; however, we note the large standard deviation in absenteeism rates, indicating that employee absenteeism is highly varied.

When compared to the literature, this workplace’s current absenteeism and presenteeism rates appear to fall within the range expressed in the literature, even with the drop in productivity between pre-pandemic and pandemic rates. For example, baseline rates of absenteeism and presenteeism (pre-pandemic) across industries were found to vary widely; Schwatka et al. (2018) found low absenteeism and presenteeism among employees of large sized enterprises (n = 1680, all industries); 12.3% had missed at least 1 h of work within the past four week period and only 21.6% reported working at their best performance. At the same time, an Australian study (n = 4593) looking at the general 18 + workforce found a mean presenteeism score of 63.61 (Johnston, 2019), somewhat comparable to our observed score of 79.78. The National Institute of Health and Occupational and Health (NIOSH) in the U.S found no significant differences in absenteeism rates during the COVID pandemic (April 2020) as compared to their 5 year baseline rates in non-essential service industries (Groenewold et al., 2020).

The apparent decline in presenteeism scores (increase in presenteeism) is likely attributable in some part to emergency remote working measures during COVID-19. One possible conjecture is that remote working offers employees’ flexibility in terms of work hours, allowing for absenteeism rates to remain stable or improve while presenteeism may increase due to distractions at home. Participants readily described the benefits of remote working, such as enhanced efficiency due to less wasted time on commuting and socializing. This is similarly reflected in our quantitative data, where respondents reported being largely in favour of remote working, and were satisfied with their workplace’s response to COVID. However, working from home is known to stretch the temporal limits of the workday, with employees deviating and working longer than their typically scheduled hours and as an adaptation to at-home distractions for carer-employees (Ding & Williams, 2022). It is also possible that remote working present challenges in communication, with managers and HR disconnected from the challenges their employees face thereby eroding the sense of workplace culture. While the HPQ scale uses contracted-hours-not-worked to measure absenteeism, it is likely the full impact of absenteeism is higher, as firms with high team-specific human capital incur costs related to reduced team productivity on top of individual absences (Zhang et al., 2017).

### OBJECTIVE 2 Do the Work and Health Outcomes of Carer-Employees Differ Significantly from Non-Carer/Regular Employees?

No significant differences between carers and non-carers in any of the health and work outcome survey variables was found, with the exception of co-worker support. Carers reported significantly less co-worker support when compared to non-carer employees. This is echoed in the qualitative interviews, where carers describe increased burdens during COVID and unsupportive work environments, leading to feelings of isolation at work. It is likely that the combination of increased carer burden and unsupportive work environments had an additive effect, creating an overall poorer perspective on support systems in the workplace, and exacerbating work–life tensions. Elsewhere in the literature, it is known that coworker supports have significant interaction effects on carer burden and work impairment, where high levels of coworker support has a buffering effect on carer burden and work impairment (Fujihara et al., 2019).

Despite this, the Kruskal–Wallis tests reflects that lower levels of coworker support for carer-employees did not translate over to health and/or work deficits (nor advantages), as compared to non-carer employees. However, results of the correlation matrices speak to the multi-modal and interconnected nature of the work experience. The significant correlation between coworker support and absenteeism signals areas of potential future deficits, if not addressed by the employer.

Overall, we observe that across the entire sample (both carers and non-carers) work-related outcome variables such as: job satisfaction, schedule control, work-family conflict, and family-work conflict, tend to be fairly average, even during COVID, and did not significantly differ between the two groups. Interestingly, while the mean score for family supportive supervisor behaviour was moderately supportive at 14.2 (across the entire sample), there is wide variation in levels of reported supervisory support; some participants rated extremely poor levels of family supportive supervisor behavior. From the interview data, carers with unsupportive supervisors disclosed that their supervisors did not improve or offer additional support with COVID, especially with the curtailing of communications into business-only. Rather, the shift to remote working buffered their interactions with their supervisors, given that employees had agency to recontract their workday around familial obligations such as caregiving when working from home. This shift in flexibility favours carer-employees and aligns with current suggested business practice models (Arksey, 2002). Prior to COVID, the majority of interventions targeting work-life balance were centered on making use of flexible working arrangements as a primary intervention. As such, it is to the benefit of carer-employees to retain some form of flexibility or remote working.

Health outcomes pertaining to self-reported health, depression and general self efficacy are also average/typical across the entire workplace sample. It is notable that, while on average the survey sample is in good health, a third of survey respondents were at risk of depression. This is an area of divergence from the interview themes, as many managers speak of mental health initiatives available for employees, despite relatively few non-manager participants knowing of these resources. It is known from the literature that a high frequency of depressive symptoms is correlated with burnout, highlighting a potential area that workplaces should pay attention to.

Although we did not find many significant differences between the carer and non-carer group, our findings are notable in that they highlight the heightened tensions that carer-employees are precariously navigating during the pandemic. Care burden likely increased as a result of modifications to care behaviour, as necessitated by COVID-19. Loss of external carer supports, such as community services and friends/family, due to stay-at-home orders and lockdowns meant that the caregiving role was experienced more singularly. In addition, carers were concerned with transmission risk as they moved between sites of work and sites of care. Overall, carers are spending more weekly time on care provision, and often in novel ways compared to pre-pandemic routines. These additional strains placed on carers are compounded by the high-stress work environment. These findings are consistent with research emerging from the UK, where carer-related anxiety, financial burden, and time spent caregiving has elevated (Carers, 2020). This area of discrepancy with non-carers is preeminent, as it has the potential to lead to future health and work complications for carers if left unchecked.

One area of dissonance observed from the qualitative findings concerned the visibility and communication of organizational supports for carers. Notably, carers communicated a lack of knowledge on what was available to them from the workplace, with none of the carer participants knowing about the internal carer network. However, managers in the interviews were able to list several supports, including EAP benefits and the carer network. This discrepancy brings attention to a disconnect in communication about these services.

### OBJECTIVE 3: How Can Workplaces Adapt to Support Their Employees, Caregiving or Otherwise?

From our findings, we identified two main gaps pertaining to future intervention design and implementation within our workplace: lack of consistent managerial support, and, lack of visible and explicit messaging regarding caregiving and/or resources available for carers. We note that the theme of unsupportive work culture, in the form of managers and coworkers, was raised concurrently in the qualitative and quantitative findings, and presents a tangible barrier to carer-employees. These findings within the context of our partnered organization raises broader questions related to the organizational readiness for change among other workplaces. That is, while our partnered workplace self-identified as interested in supporting carer-friendly workplace initiatives, we still observe room for improvement regarding their work culture. It is conceivable that other organizations that are less carer-friendly, may need to address additional and intensified barriers in culture and managerial attitudes when designing workplace interventions.

We acknowledge that the pandemic facilitated the widespread uptake and acceptance of remote working, and thus removed the urgency for workplace interventions during COVID to address flexibility. Instead, we suggest centering campaigns on improving workplace culture. Virtual workplaces and teams often lack opportunities for team bonding, leading to diminishment of social cohesion, trust and support (Newman & Ford, 2021). COVID further disconnects employees, which leads to underlying anxieties specific to: individual and public health, finances, physical distancing mandates, as well as general uncertainty left in the wake of the pandemic. This disconnect in workplace culture espouses insular and individualized experiences with paid employment (Kniffin et al., 2021). The targeting of leadership and visibility of work-life initiatives for carers thereby sends a message of organizational commitment and support throughout crisis situations, as well as throughout employees’ life trajectory.

We propose the following interventions:

1. An educational visibility campaign promoting workplace carer policies
2. Standardized training of managers

These recommendations follow the CSA’s current workplace standard for carers, where building awareness and establishing competency of leaders is a foundational step towards cultivating a carer-friendly workplace (CSA, 2017).

Carers would benefit from a visibility campaign aimed at: increasing the profile of carer-employees within the workplace, promoting existing policies and resources, and visibility committing organizational support to carer issues. This intervention may manifest in the form of email alerts, virtual and physical posters/banners, message board announcements, social media alerts, and pre-meeting announcements. Lunch and learn seminar sessions may be offered to any interested employees during lunch breaks, highlighting carer statistics, as well as the resources available in the workplace, at the provincial and federal scales. In our case, this process is facilitated by the fact that our partnered workplace already contains numerous internal supports and policies for carers, such as: an internal carer network, EAPs, and several leaves. As a result, the focus should be on bringing attention to these existing services.

In organizations that lack such policies for carers, the initial step should be the review, update, and creatation such policies. Visibility or informational interventions – with a focus on building awareness to induce behavioral and attitude change – are often low cost and correspondingly, easily implemented as they utilize existing infrastructure. Information-based interventions in the workplace are generally effective, although with small-to-moderate effect sizes; Bellon et al. (2019) found in their meta-analysis of three randomized control trials, that psychosocial and educational interventions in the workplace were capable of significantly reducing depression risk of employees. Other systemic reviews found that approximately 70% of psychosocial and educational interventions (N = 23) designed for improving workplace safety report positive impacts (Aburumman et al., 2019). Few studies directly assess workplace interventions for carer-employees; in a previous pilot study from our own research program that was conducted at a university workplace, we found evidence that informational interventions were capable of significantly improving health and work outcomes of carer-employees (Ding et al. 2020, 2021).

However, given that organizational culture is often deep-rooted to the inherent leadership structure of a workplace, we propose that this visibility intervention component runs concurrently to a supervisory training component. Supervisory training should contain mandatory sessions, run by a trained workplace champion, educating managers/supervisors on: changing demographic trends, economic contributions of carers, carer burden and health, legal obligation of employers, as well as available federal and provincial resources, and empathetic/compassionate approaches to work-family conflict. Training sessions should also include role-playing through different potential situations, accompanied by a guidebook document. Managerial attitudes and support of their employees is crucial to forming workplace culture, to the extent that employees may mirror supervisory work-life balance behaviour. Thus, training sessions for managers have the capacity to induce wider culture change by changing or increasing discourse on work-family conflicts. Unsupportive managerial attitudes have been known to cause reluctance in employees’ utilization of formal workplace supports and accommodations, harming carer-employee job satisfaction and putting this cohort at risk of burnout, psychological distress and turnover (Crain & Stevens, 2018).

Organizational culture change is highly context dependent; qualitative studies find that successful workplace culture change interventions centered around mental health promotion are contingent on a number of specific organizational elements, such as support from leadership, affirmative dialogue, and positive working group dynamics (Knaak et al., 2019). It is notable and of interest that in our environmental scan, employees (caregiving and non-caregiving) generally reported wide variations in family-supportive supervisor behaviours during COVID-19. While supervisors interviewed within our study were generally supportive of caregiving responsibilities in general, as well as being knowledgeable of supports and accommodations in the workplace, there is evidently a breakdown in communication elsewhere, as the carers themselves were unaware of the said policies, and some had negative experiences with their direct supervisors. Managers are often transformative agents within the workplace, as they exercise authority over: the types of support employees may receive, delivery of information from executives, and implementation of work-life initiatives, thereby dictating organizational culture on a day-to-day level (Straub, 2012). Generally, employees who report their supervisors as empathetic to work-family issues, tend to have increased coping ability, productivity, team functionality, and loyalty to the workplace, in addition to lower levels of anxiety, depression, and work-family conflict (Straub, 2012). From our own correlation matrix, we observe family supportive supervisor behaviour is positively correlated with job satisfaction. This is supported by evidence from the literature, where the strong association between supervisory support of work-family-life matters and job satisfaction is well established (Crain & Stevens, 2018). In this manner, the training and education of leadership personnel to be more carer-supportive compliments the effects of the visibility campaign and offers the overall intervention a better chance of success in this workplace. Within the context of COVID, it is even more crucial for managers and supervisors to display empathy and support towards work-family conflicts, as many employees (caregiving or otherwise) are navigating heightened tensions and burdens.

## Limitations

All findings should be interpreted within the limitations of the study. First and foremost, our sample size for both the survey and interview components were small and, as such, limited in scope. Recruitment for participants was hampered by COVID regulations in force at the time of data collection, where most employees had recently shifted to working from home and navigating new work dynamics and technologies. We hypothesize that due to these (at the time) recent events, recruitment emails were lost among the shuffle. This may mean that our survey sample may not be an accurate representation of the overall workforce. In addition, we recognize that generalizability of our findings are limited, given that our respondents from the partnered workplace was largely highly educated, well-paid, and had relatively little cultural diversity. These findings may not be applicable to other workplaces with a different demographic makeup or different employee profile. As well, given the turbulent global environment at the time of data collection, it is not certain that observed changes in quantitative measurements are the result of the pandemic directly. As a result, it is difficult to extrapolate these findings beyond the context of the COVID pandemic.

## Conclusion

The COVID-19 pandemic has demonstrated not only shifts in the work landscape, but the changing needs of the labourforce, particularly for carers that are navigating the dual burden of unpaid caregiving and employment. In our present study, we find that the work experience during the pandemic has fundamentally changed, for both carers and non-carers. As most employees have been working from home, the work experience has become more isolating, leading to a disconnect from colleagues and the organizational culture, as well as increases in presenteeism. However, at the same time, employees report greater schedule control and flexibility as a function of remote working, which is advantageous for carer-employees in balancing care demands. While care demands and conflict are greater during the pandemic, most carer-employees (and non-carer employees) were found to be in good health, despite additional/shifting care responsibilities, such as more frequent phone calls. Overall, we note that health and work outcomes do not different significantly between carer and non-carer employees, except for coworker support, where carer-employees report significantly less support.

From our findings, we identified two major areas that are pertinent to future intervention design and implementation. The first pertains to visibility and consistency of messaging around workplace supports and resources for caregiving and work-family conflicts. Managers and HR personnel were unable to identify said resources, while carer-employees were generally unaware, indicating a breakdown of communication. Second, survey results and some participant experiences depicted a wide variation in family supportive supervisor behaviour, signally inconsistency in managerial attitudes and approaches to work-family conflict. These findings set the stage for future workplace interventions going forward. Based on our findings, we propose that within this workplace, interventions should contain visibility/educational campaigns, in the form of posters, email announcements, as well as announcements at the start of meetings, highlighting carer statistics as well as existing resources. Concurrently to this, managers should undergo standardized training on carers in the labour force, with an emphasis on compassionate language, legal obligations, and available resources within the workplace and community. In doing so, we hope that such interventions are capable of promoting carer-inclusive and carer-supportive workplace culture, benefitting both carer-employees and employers. For policymakers and employers, we hope that our research demonstrates the importance of specific and context-driven intervention design within workplaces.

## Carer-Employee Interview

[Hand out information regarding the Standard highlights. Give few minutes to look over.] 1. Why did you decide to join this research?

### Caregiving Prompts

2. Has your caregiving behaviour changed since COVID-19? a. PROBE: changes in time spent caregiving, changes in activities b. What challenges have you encountered while caregiving during COVID-19? (i) Do you see these challenges as being long-term challenges? PROBE: Do you anticipate returning to your “normal” routine? c. Do you see the carer role being transformed permanently by COVID-19? Why or why not?

### Work Prompts

3. How has your paid work responsibilities been affected by COVID-19? Have demands on your time changed? How about your workload? Has there been any changes in work flexibility? a. Were you satisfied with your work situation before COVID-19? (i) PROBE: flexibility, workload, demands on time b. Are you satisfied with your current work arrangements and supports during COVID-19? c. Would you like to see these arrangements continue to be offered after COVID-19? Would you consider using these arrangements again in the future? 4. Have you noticed changes in workplace culture since COVID-19? In terms of co-workers? Supervisors? a. PROBE: communication, supports, use of benefits, flexible work arrangements

### Intersection of Care and Work Prompts

5. Are you currently finding it difficult to balance paid work, caregiving, and personal time? a. PROBE: current and future difficulties 6. What would you like to see being implemented in your current workplace to help you as a carer-employee? a. What have you found to be most useful in helping you manage or cope as a carer? b. What have you found to be most useful in helping you manage as an employee?

### CSA Standard Prompts – Refer Again to Standard Summary Document/Highlight

7. Do you see the Standard helping you manage your work? Your caregiving life? What are benefits of the Standard becoming available for use by employees in general? a. Probe: comparison of benefits to workplace vs benefits of employees if respondents do not discuss first themselves 8. In your experience, what supports/services/accommodations, what aspect of the Standard do you think are most useful to you as a carer? a. What are the least useful? Why? 9. What would you add or improve about the Standard to make it better? 10. Do you see this Standard being useful in all situations? Are they any situations in which the Standard cannot cover? Take into account social, cultural, ethnic lenses. 11. Are there any concerns about implementation of this Standard?

Is there anything we forgot or is there something important that we should know?

## Key Informant Interview

[Hand out information regarding the Standard highlights. Give few minutes to look over.] 1. Why did you decide to join this research?

### Current Workplace Prompts

2. What do you personally consider as the priorities of your organization? This can be in terms of the actual work that you do, your employees, or other societal aspects. 3. Are you satisfied with your workplace’s organizational culture at the moment? a. Are there any changes that you would like to see made? 4. Are there policies, programs or supports in place, that you are aware of for your employees to support work-life balance? Are there any specific to employees who are currently caregiving? a. Do you see these services as adequate? What about in the coming years?

### COVID-19 Prompts

5. What measures has your workplace/department taken to in response to COVID-19? a. Are these measures sustainable in the long-term? b. Are these arrangements likely to be continued to be offered after COVID? 6. Carer-employees must provide care to a loved one for health, disability or age-related reasons while maintaining employment obligations. Do you think the dual burden shouldered by CEs is a concern at the moment to your organization? 7. Given that the COVID-19 pandemic is impacting the elderly and immunocompromised populations severely, have you made any arrangements for employees juggling caregiving on top of employment? Why or why not? a. Has COVID-19 changed your perception on the challenges of the carer-employee role? b. How important do you think it is to your organization to support employees who are currently juggling caregiving and employment? What about employees who may “juggle” in the future? c. Would you provide supports for employees who are caregiving after COVID-19?

### Standard Prompts – Refer to Standard Summary/Highlight Document

8. Where do you see the Standard fitting in with your organizations’ goals mandate? 9. In what ways do you see the helping your organization? What are the anticipated benefits to implementation of the Standard? Probe: organizational efficiency and work-life balance as priorities 10. What aspects of the Standard do you think are most useful to you? To your employees? a. What are the least useful? Why? 11. What would you add or improve about the Standard to customize it to your organization? a. Do you foresee any situation in which the Standard is not adequate? b. Are there any situations in which the Standard will not apply? Ex. Religious events, cultural forms of care Probe: limits to the Standard, COVID-19 12. Are there any concerns about implementation of this Standard? 13. In the future, do you foresee the process of implementing/changing employee programs (ex. Benefits, accommodations, campaigns) being affected due to COVID-19? In what ways?

Is there anything we forgot or is there something important that we should know?

## Appendix C

Convergent mixed method triangulation of qualitative findings with quantitative results.

**Table:**

Component	Quantitative	Qualitative	Triangulation
Workplace Context	Large-sized workplace (> 3500 employees), highly educated, mean hourly wage of $43.01/hour		Qualitative (Silence)
Carer experience	50% of carers have high carer burden (Zarit Burden > 17)	Carers experiencing additional burdens managing carework and paid work during COVID	Convergent
	Weekly care hours increased with COVID-19	Additional caregiving challenges introduced by COVID-19 such as concerns with COVID transmission, phone calls, lack of external resources	Convergent
	Carers report signficantly reduced levels of coworker support as compared to non-carers (p > 0.017)		(Qualitative) Silence
		Carers have lack of knowledge on existing carer policies	(Quantitative) Silence
Work experience	Generally good health, with 50% of the survey sample with CES-D scores indicating risk of depression (CES-D > 10)	High workload due to workculture emphasis on bilability, leading to burnout; increased work demands due to layoffs	Convergent
	Absolute presenteeism 79.8	New teleconferencing technology to use; Challenges with virtual collaboration	Convergent
	Absolute absenteeism is 12.1		Qualitative (Silence)
	Rated family supportive supervisor behavior overall moderately supportive, at a mean of 14.1 (SD 4.28) across the survey sample, with large range in scores	Wide variation in participant experiences with supervisors; some participants feel unsupported by their supervisor, while others feel very supported; Supervisors were knowledgable in available supports in the workplace for carer-employees	Convergent
		Carers were not aware of many supports and programs within the workplace	Dissonance
COVID-19	High level of schedule control (mean of 30.2)	Majority of employees currently working-from-home, increased flexibility	Convergent

## Footnotes

1 Schmitz, A., Quashie, N. T., Wagner, M., & Kaschowitz, J. (2022). Inequalities in caregiving strain during the COVID-19 pandemic: conceptual framework and review of the empirical evidence. *International Journal of Care and Caring*, 1-14.
